# Global knowledge graph of osteoporosis biomarkers based on large language model embeddings and complex network algorithms

**DOI:** 10.3389/fendo.2026.1776707

**Published:** 2026-05-08

**Authors:** Qizhi Tan, Wenchu Liang, Manting Liu, Qiaoming Fan, Wenhao Pan, Junlin Zhou, Qingyi Yang, Kexin Wang

**Affiliations:** 1 Guangzhou University of Chinese Medicine, Guangzhou, China; 2 The Eighth Clinical Medical College of Guangzhou University of Chinese Medicine, Foshan, China; 3 Guangdong Provincial Hospital of Integrated Traditional Chinese and Western Medicine, Foshan, Guangdong, China

**Keywords:** biomarkers, complex network analysis, knowledge graph, large language model embeddings, osteoporosis

## Abstract

**Background:**

This study applies advanced artificial intelligence technologies to reconstruct the global research landscape of osteoporosis (OP) biomarkers and to predict emerging nodes with potential clinical value.

**Methods:**

Literature from the Web of Science Core Collection and PubMed over the past 20 years was integrated. The OpenAI text-embedding-3-large model mapped keywords into a high-dimensional semantic space, enabling deep clustering through cosine similarity and reducing polysemy. The Walktrap community detection algorithm and Callon strategic diagram were used to identify high-centrality, low-density themes in the “fourth quadrant.” Gemini 2.5 Pro was applied to generate entity–relation triples, which were validated through a human-in-the-loop process and integrated into a refined knowledge graph. Topological indicators such as betweenness centrality were used to detect emerging potential nodes.

**Results:**

A total of 1595 core publications were included, showing a significant linear rise in annual output. Nine major research themes were identified. The strategic diagram highlighted three high-potential domains: inflammation and immune regulation, detection technologies and biosensing, and gut microbiota and metabolism. Knowledge-graph analysis revealed oxidative stress as a key bridging node integrating immune, metabolic, and other systems. Several emerging nodes with strong connective potential were identified outside traditional hotspots, including molecular biomarkers such as lncRNA MEG3, miR-21-5p, spermidine, and Treg-Exo, as well as imaging innovations such as opportunistic CT screening and vertebral bone marrow PDFF. These findings indicate a paradigm shift in OP research from single-hormone mechanisms toward a multidimensional network involving inflammation, immunity, gut microbiota, and imaging.

**Conclusion:**

Integrating AI-based embedding models with complex network algorithms, this study overcomes limitations of traditional bibliometrics and systematically uncovers the latent knowledge structure of OP biomarkers. The identified emerging nodes offer robust evidence and forward-looking guidance for early precision screening-such as liquid biopsy and radiation-free imaging-and for developing novel therapeutic strategies, including bone-targeted exosomes and gut–bone axis interventions.

## 1 Introduction

Osteoporosis (OP) is a systemic skeletal disorder characterized by reduced bone mass and deterioration of the bone microarchitecture. These alterations lead directly to increased bone fragility and heightened susceptibility to fractures. As an age-associated degenerative condition, OP and its related fragility fractures rise sharply in prevalence alongside global population aging. Epidemiological evidence indicates that women exhibit a markedly higher prevalence than men, with postmenopausal women being particularly affected (1). After reaching peak bone mass, bone quantity declines naturally at an annual rate of approximately 0.5%. The pathogenesis of OP is highly complex. It involves multiple factors, including cellular senescence, hormonal dysregulation (such as vitamin D and parathyroid hormone), abnormalities in growth factors such as FGF-23, chronic inflammatory conditions, altered mechanical loading, and genetic susceptibility (2). Notably, recent studies have demonstrated that sclerostin, a protein secreted by osteocytes, negatively regulates osteoblast function. It markedly suppresses bone formation and has emerged as a key pathological mechanism (3). As global aging accelerates, the socioeconomic burden imposed by OP is expected to continue increasing. Therefore, early and precise diagnosis, together with long-term monitoring of high-risk populations, is essential for reducing OP-related risks and fracture incidence.

In parallel, artificial intelligence (AI) and machine learning (ML) have rapidly expanded across biomedicine, reshaping approaches to disease screening, diagnosis, and knowledge discovery. Recent bibliometric analyses indicate an exponential increase in AI and machine learning applications in biomedical research over the past decade, particularly in imaging, genomics, and precision medicine. In OP research, AI-driven approaches have been applied to multimodal datasets including DXA, CT, MRI, and electronic health records, enabling more accurate bone mineral density prediction, fracture risk assessment, and phenotype stratification (4–7). These trends underscore the growing value of intelligent analytic methods in extracting clinically meaningful information from complex biomedical data, providing a strong rationale for the present study.

Driven by advances in AI-enabled biomedical analytics, real-world evidence, and basic medical research, the discovery of OP biomarkers has expanded rapidly in recent years. Recent OP research has concentrated on several key areas. Imaging technologies, including DXA, CT, and MRI, remain central for bone mineral density evaluation and fracture risk assessment. Emerging molecular biomarkers, such as non-coding RNAs (e.g., lncRNA MEG3, HOTAIR) and microRNAs (e.g., miR-21-5p), have shown potential for early detection and mechanistic insights. Immunoregulatory pathways and gut microbiota interactions are increasingly recognized for their role in bone metabolism. Despite these advances, the literature remains fragmented, and traditional bibliometric approaches have focused mainly on established hotspots, often overlooking emerging but highly connected nodes. In this context, our study leverages AI-driven semantic embeddings and complex network analysis to construct a global knowledge graph of OP biomarkers, enabling the identification of frontier themes and potential nodes that are not yet widely recognized, thus representing the main innovation of this work (8, 9).

However, the field still faces substantial challenges. First, the number of publications is increasing exponentially, and research findings are becoming highly fragmented. Consequently, it is difficult for researchers to systematically identify biomarkers with the greatest potential for clinical translation from massive and heterogeneous datasets. Second, traditional bibliometric approaches rely heavily on exact matching of keywords. Such methods struggle to detect core concepts that are semantically related yet expressed differently, for example, “bone loss” and “osteopenia.” This limitation restricts both the breadth and depth of frontier identification and leads to information omission. Third, existing reviews tend to focus on well-established research hotspots. They rarely quantify or predict “emerging nodes,” which remain in their infancy yet possess high connective potential within the scientific network.

To overcome these limitations, this study introduced advanced artificial intelligence techniques and complex network algorithms. Unlike traditional frequency-based analytical paradigms, we employed the high-performance text-embedding-3-large model developed by OpenAI. This model mapped complex biomedical keywords into a high-dimensional vector space. Deep semantic clustering was achieved through cosine similarity, which effectively resolved issues related to polysemy and synonymy in terminology. In parallel, the Walktrap community detection algorithm and the Callon strategic diagram were incorporated. These tools enabled not only a macro-level reconstruction of the global research landscape on OP biomarkers but also the precise identification of frontier themes located in the fourth quadrant. These themes exhibit high centrality and close associations with core domains despite their still-developing internal structure (10).

Importantly, unlike most current AI-related OP studies that focus mainly on image-based diagnosis, fracture prediction, or genomic risk modeling, the present study applies AI-driven semantic mining to the biomarker knowledge system itself. By integrating large-model semantic embeddings with complex network analysis, this work moves beyond simple keyword co-occurrence and enables the identification of semantically related, translationally relevant, and still-emerging research nodes within a rapidly expanding literature corpus.

Building upon this framework, the study integrated data from both the Web of Science and PubMed databases to construct refined knowledge graphs for three high-potential domains: inflammation and immune regulation, detection technologies and biosensing, and gut microbiota and metabolism. Key topological metrics, including betweenness centrality, were computed to identify a series of emerging potential nodes. Through this data-driven approach, the study aims to provide systematic evidence and forward-looking insights for early OP screening strategies, precision diagnostic classification, and the development of new therapeutic targets.

## 2 Methods

### 2.1 Data sources

The primary data source for this study was the Web of Science Core Collection (WoSCC) database, which was utilized to extract bibliometric indicators. These indicators included publication volume, citation frequency, keyword frequency, and total link strength (TLS). To validate the robustness and generalizability of the Walktrap clustering algorithm, the PubMed database was incorporated as an external validation set. Additionally, regional Human Development Index (HDI) data were sourced from the latest statistical datasets released by the United Nations Development Programme. These data quantify the comprehensive achievements of various countries and regions across key dimensions of human development.

All data used in this study were obtained from publicly available, de-identified datasets from Web of Science Core Collection and PubMed. No individual patient-level data were accessed or analyzed. Ethical considerations were carefully addressed by ensuring that all sources complied with relevant data privacy regulations and publication standards. The study adhered to principles of responsible data use, transparency, and reproducibility in large-scale biomedical research. All analyses were conducted in accordance with institutional guidelines and international best practices for the ethical handling of bibliometric and literature-derived data.

### 2.2 Search strategy and inclusion criteria

The search strategy was developed in strict accordance with the combination of Medical Subject Headings (MeSH) and free-text terms. Data screening was performed using the advanced search function within the WoSCC database. The search strings encompassed terms related to OP and its relevant subtypes—including senile OP, postmenopausal OP, and disuse OP—as well as biomarkers. The specific query was: (TS=“Osteoporoses”) OR (TS=“Osteoporosis, Age-Related”) OR (TS=“Osteoporosis, Age Related”) OR (TS=“Age-Related Osteoporosis”) OR (TS=“Age-Related Osteoporoses”) OR (TS=“Age Related Osteoporosis”) OR (TS=“Osteoporoses, Age-Related”) OR (TS=“Bone Loss, Age-Related”) OR (TS=“Age-Related Bone Loss”) OR (TS=“Age-Related Bone Losses”) OR (TS=“Bone Loss, Age Related”) OR (TS=“Bone Losses, Age-Related”) OR (TS=“Osteoporosis, Senile”) OR (TS=“Osteoporoses, Senile”) OR (TS=“Senile Osteoporoses”) OR (TS=“Senile Osteoporosis”) OR (TS=“Osteoporosis, Involutional”) OR (TS=“Osteoporosis, Post-Traumatic”) OR (TS=“Osteoporosis, Post Traumatic”) OR (TS=“Post-Traumatic Osteoporoses”) OR (TS=“Post-Traumatic Osteoporosis”) AND biomarker (including its free-text variants). The search period spanned from the inception of the database to October 1, 2025.

To ensure the quality and reliability of the included literature, strict exclusion criteria were established: (1) non-English language publications, as language barriers may restrict the global dissemination of research findings; (2) non-peer-reviewed document types (e.g., editorials, news reports, errata, book reviews, and meeting abstracts), whose scientific rigor cannot be guaranteed due to the absence of a stringent peer-review process (11). The final inclusion was limited primarily to original research articles and reviews.

### 2.3 Data collection and keyword standardization

Data collection was independently conducted by two rigorously trained researchers. Any discrepancies were resolved through discussion or by the decision of a third senior researcher.

Keywords are pivotal for elucidating the core content of research themes. However, owing to variations in author terminology and the heterogeneity of indexing algorithms across databases, identical concepts often manifest in diverse forms (e.g., “Bone Loss” versus “Osteopenia”). Traditional approaches relying on spelling similarity or lemmatization are often inadequate to address this issue of semantic heterogeneity. To address this, an innovative semantic clustering method combining “Embedding + Cosine Similarity” was introduced for standardization.

**Figure 1:**
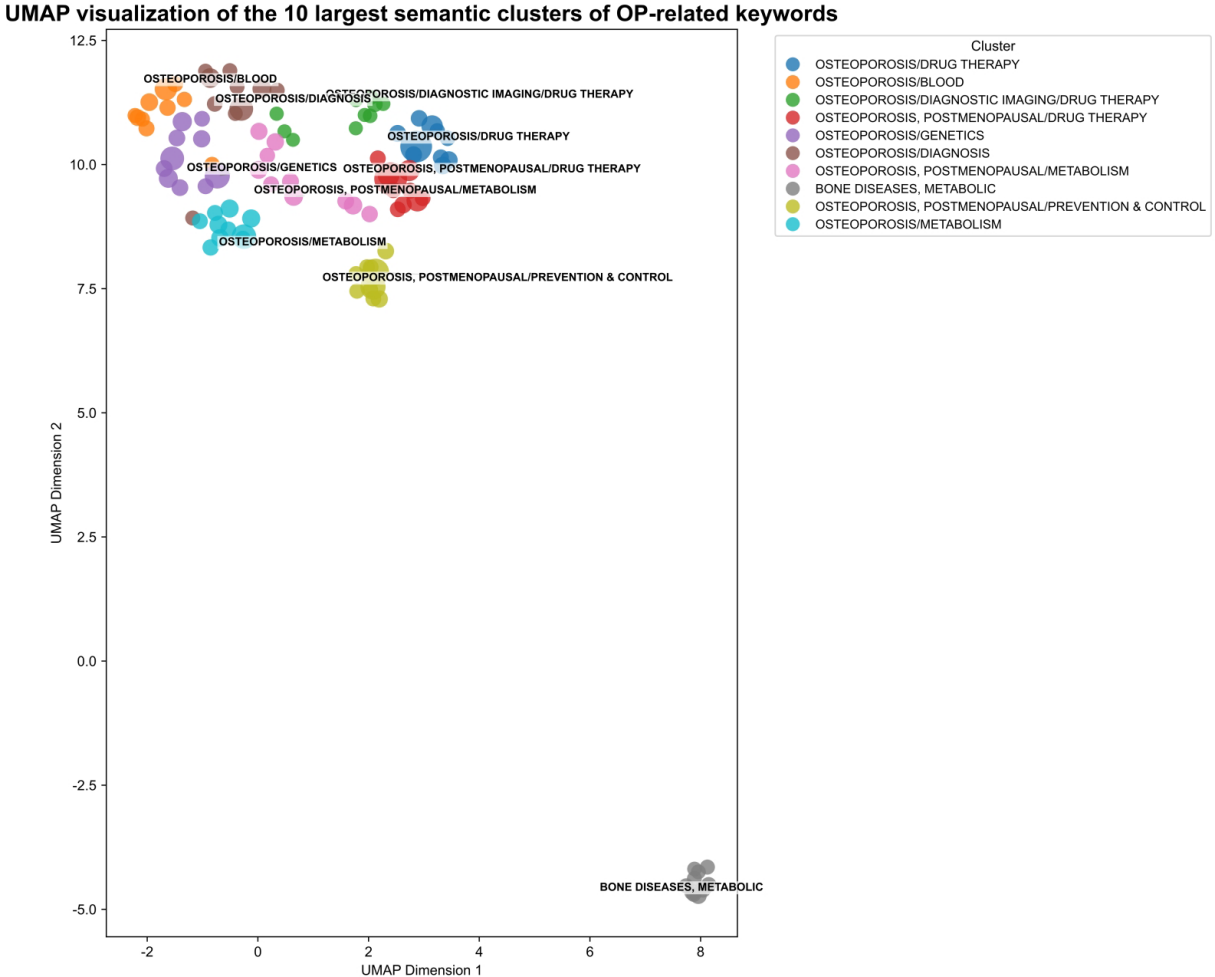
UMAP visualization of the 10 largest semantic clusters of osteoporosis-related keywords. For each cluster, the 10 highest-frequency keywords were selected for visualization. The size of each point is proportional to the keyword frequency, and labels denote representative (canonical) keywords. Colors indicate cluster membership.

The specific workflow was as follows: First, all keywords retrieved from WoSCC and PubMed were merged. Subsequently, the keywords were mapped into a high-dimensional vector space utilizing the text-embedding-3-large model developed by OpenAI. For example, a set of keywords related to “bone turnover markers” including “Osteocalcin,” “CTX-I,” and “P1NP” were embedded using the text-embedding-3-large model into high-dimensional vectors. The cosine similarity between each pair of vectors was calculated, and a similarity threshold of 0.85 was applied to group semantically related terms into clusters. Within each cluster, the most frequent term was selected as the canonical keyword. This process effectively consolidated synonyms and variants, reducing semantic redundancy. A schematic of this embedding and clustering workflow is illustrated in **Figure 1**, highlighting the transformation from raw keywords to standardized semantic clusters.

All keywords retrieved from WoSCC and PubMed were merged and mapped into a high-dimensional vector space using the OpenAI text-embedding-3-large model. Pairwise cosine similarity was computed for all keyword vectors. A similarity threshold of 0.85 was selected based on empirical tuning to balance cluster granularity and semantic coherence: higher thresholds produced overly fragmented clusters, while lower thresholds merged semantically distinct terms. Keywords with cosine similarity above the threshold were grouped into clusters. Within each cluster, the most frequent keyword was selected as the canonical keyword for downstream analysis. This process reduces semantic redundancy while preserving biologically meaningful clusters.

### 2.4 Bibliometric analysis and network mapping

Bibliometrics effectively tracks the evolutionary trends of a discipline through the quantitative analysis of literature characteristics, such as publication volume, authors, journals, and citation networks (12, 13). Bibliographic information was extracted from full records exported from WoSCC. HistCite (v12.03.17) was utilized to calculate the Total Global Citation Score (TGCS), which served as a core indicator for assessing research impact.

Network mapping analysis primarily encompassed co-authorship analysis, co-occurrence analysis, and co-citation analysis. Co-authorship analysis was employed to delineate scientific collaboration networks among countries, institutions, and authors (14). Keyword co-occurrence analysis quantified association strength by calculating the frequency with which terms appeared jointly in the same document, thereby visualizing current research hotspots. Co-citation analysis facilitated the exploration of the intellectual base of the field (15). In the network visualization maps, node size corresponded to frequency of occurrence or citation count, while color denoted the assigned cluster. The thickness and length of the connecting lines indicated the strength of association and correlation between nodes.

### 2.5 Topic clustering based on Walktrap algorithm and strategic diagram

Based on the standardized keyword data, a keyword co-occurrence matrix was constructed. To ensure the connectivity of the network topology and the validity of the analysis, isolated nodes were removed, and only the giant component was retained. Subsequently, the Walktrap algorithm was employed to cluster the network. This algorithm, grounded in the principle of random walks, exploits the tendency of short random walks to remain within high-density communities. By calculating random walk distances between nodes, it effectively identifies dense subgraphs-representing research topic clusters-within complex networks.

Building upon the clustering results, a strategic diagram model was introduced to quantify the developmental status of each topic cluster by calculating two dimensions: centrality and density. Density (Y-axis) characterizes the strength of internal links within a cluster, reflecting the internal cohesion and maturity of the topic. Centrality (X-axis) characterizes the strength of links between a specific cluster and other clusters, reflecting the core position of the topic within the entire research network and its relevance to other fields.

The diagram categorizes topics into four quadrants. The fourth quadrant, designated as Basic/Future Themes, is characterized by “low density and high centrality.” Although the internal structure of these topics requires further refinement, they maintain close connections with core research areas. Consequently, they typically represent foundational research directions or frontier trends with high potential. This study places specific emphasis on this quadrant.

Network density was calculated as the ratio of the number of observed edges to the maximum possible number of edges in the undirected graph, i.e., *Density* = 2*E*/(*N*(*N*−1)), where *E* is the total number of edges and *N* is the number of nodes. Node-level centrality metrics were computed using the NetworkX Python library and included: degree centrality (number of connections per node normalized by maximum possible connections), betweenness centrality (fraction of all shortest paths passing through a node), closeness centrality (inverse of the average shortest path distance from a node to all others), and eigenvector centrality (relative influence of a node based on its neighbors’ centralities). These metrics were used to identify hub nodes, bridge nodes, and to quantify the overall connectivity of the knowledge graph.

### 2.6 High-potential knowledge graph construction

A refined knowledge graph was constructed specifically for the high-potential areas identified in the fourth quadrant of the strategic diagram. Initially, the full texts of relevant literature were retrieved for secondary screening. Subsequently, the Gemini 2.5 Pro large language model was utilized to perform automated information extraction based on a triplet format: “Entity A (Biomarker) - Relation B (Regulation/Association) - Entity C (Pathological Outcome/Mechanism)”.

During human-in-the-loop verification of extracted entities and relationships, a random stratified sample of 5% of the annotations from each cluster was independently reviewed by two evaluators. Stratification ensures proportional representation across high-frequency and low-frequency clusters. Inter-rater agreement was quantified using Cohen’s kappa coefficient, with values above 0.80 indicating strong agreement. Discrepancies within the sampled subset were resolved through discussion, or adjudication by a third senior reviewer if consensus could not be reached.

Finally, topological indicators of the nodes were calculated, including degree centrality, betweenness centrality, and closeness centrality. Bridge nodes were defined as nodes connecting two or more distinct domain clusters, revealing cross-system regulatory hubs. Emerging potential nodes were defined as nodes with “degree centrality below the median” but “top-ranked betweenness centrality.” While these nodes are not currently mass hotspots (indicated by low connectivity), they act as critical “gatekeepers” or intermediaries in the flow of network information, presaging research directions for the next generation of biomarkers.

Triplet extraction from biomedical text was performed using a large language model (LLM). Prompts were carefully engineered to instruct the model to identify subject, predicate, and object entities from each sentence. A typical prompt included: “Given the following sentence, extract all subject-predicate-object triplets in JSON format. Sentence: <input_text>“. Input texts were abstracts, titles, or sentences from full-text articles. The output was returned as JSON with fields subject, predicate, and object. Post-processing scripts validated the output, removed duplicates, and standardized entity names using controlled vocabularies (e.g., MeSH terms for biomedical concepts).

During human-in-the-loop validation of extracted entities and relationships, a random stratified sample of 5% of the annotations from each cluster was independently reviewed by two evaluators. Stratification ensures that all clusters, including low-frequency and high-frequency ones, are proportionally represented. Inter-rater agreement was quantified using Cohen’s kappa coefficient, with values above 0.80 indicating strong agreement. Any discrepancies within the sampled subset were resolved by discussion or adjudication by a third senior reviewer. This sampling-based approach enables rigorous validation while maintaining feasibility given the dataset size. Detailed guidelines and example annotations are provided in the **Supplementary Materials**.

### 2.7 Software applications and statistical analysis

Data statistics and plotting were primarily performed using the R language (version 4.3.2) and its extension packages, including rms, ggplot2, and bibliometrix. Complex network analysis was conducted using the networkx and matplotlib libraries in Python. Visualization and aesthetic refinement of network maps were executed using VOSviewer (v1.6.20) and Cytoscape (v3.10.1). To ensure the reproducibility of all analyses and figures presented in this study, the code scripts and raw data are provided in the **Supplementary Materials**.

## 3 Results

### 3.1 Global trends in publication output

**Figure 2:**
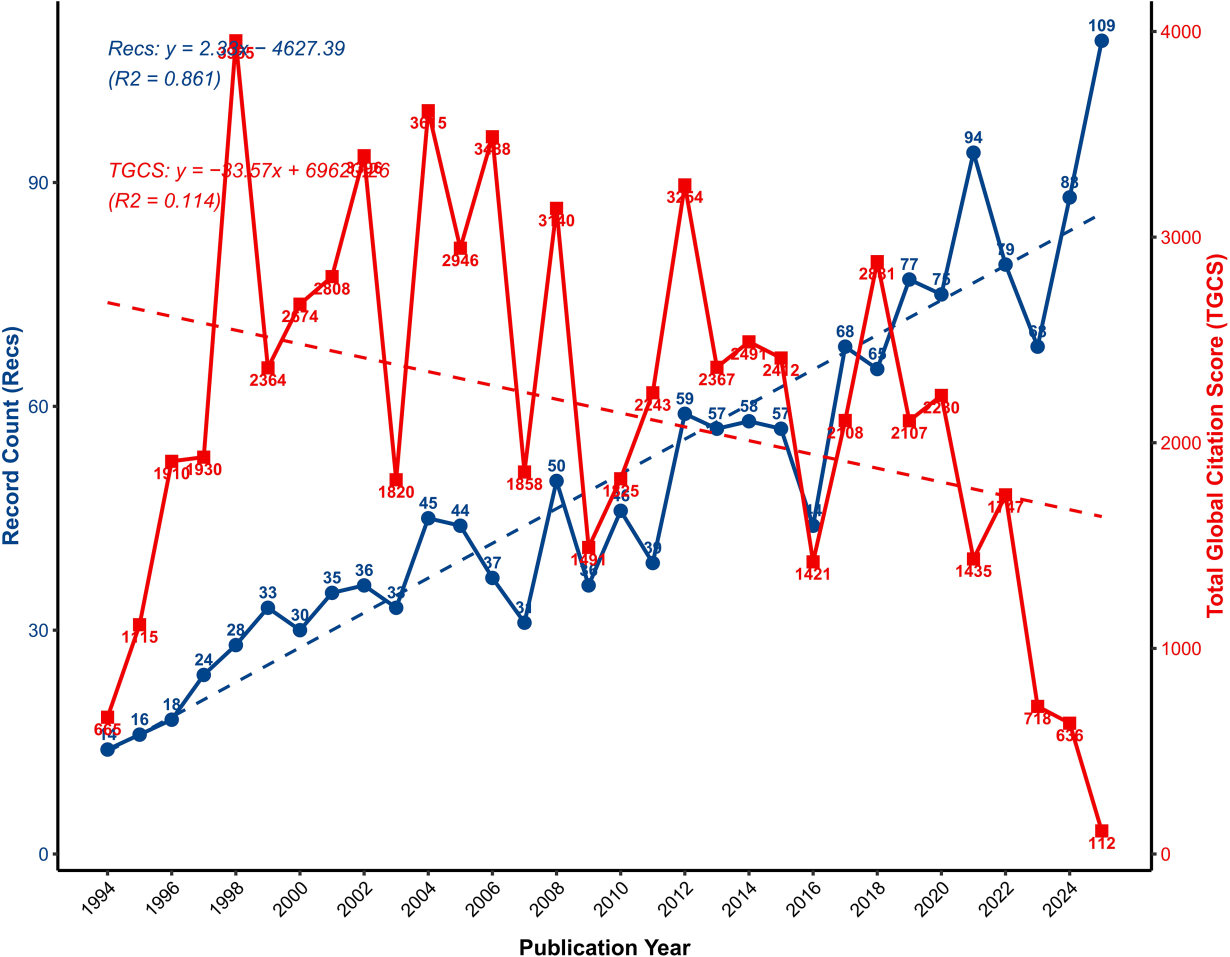
Annual publication counts and TGCS trends in global OP biomarker research.

Based on search results from the WoSCC database, a total of 1,595 documents related to OP biomarkers were identified and included in the analysis. Over the past two decades, annual publication volume exhibited a significant linear growth trend (fitting curve: Y = 2.33x−4627.30). This trajectory indicates a year-on-year escalation in academic interest within this field. Notably, the last five years witnessed a rapid surge in publication volume, rising from 68 articles in 2020 to 109 in 2025. This surge suggests that the field is currently undergoing a phase of rapid development (**Figure 2**).

### 3.2 Distribution of journals and highly cited articles

**Figure 3:**
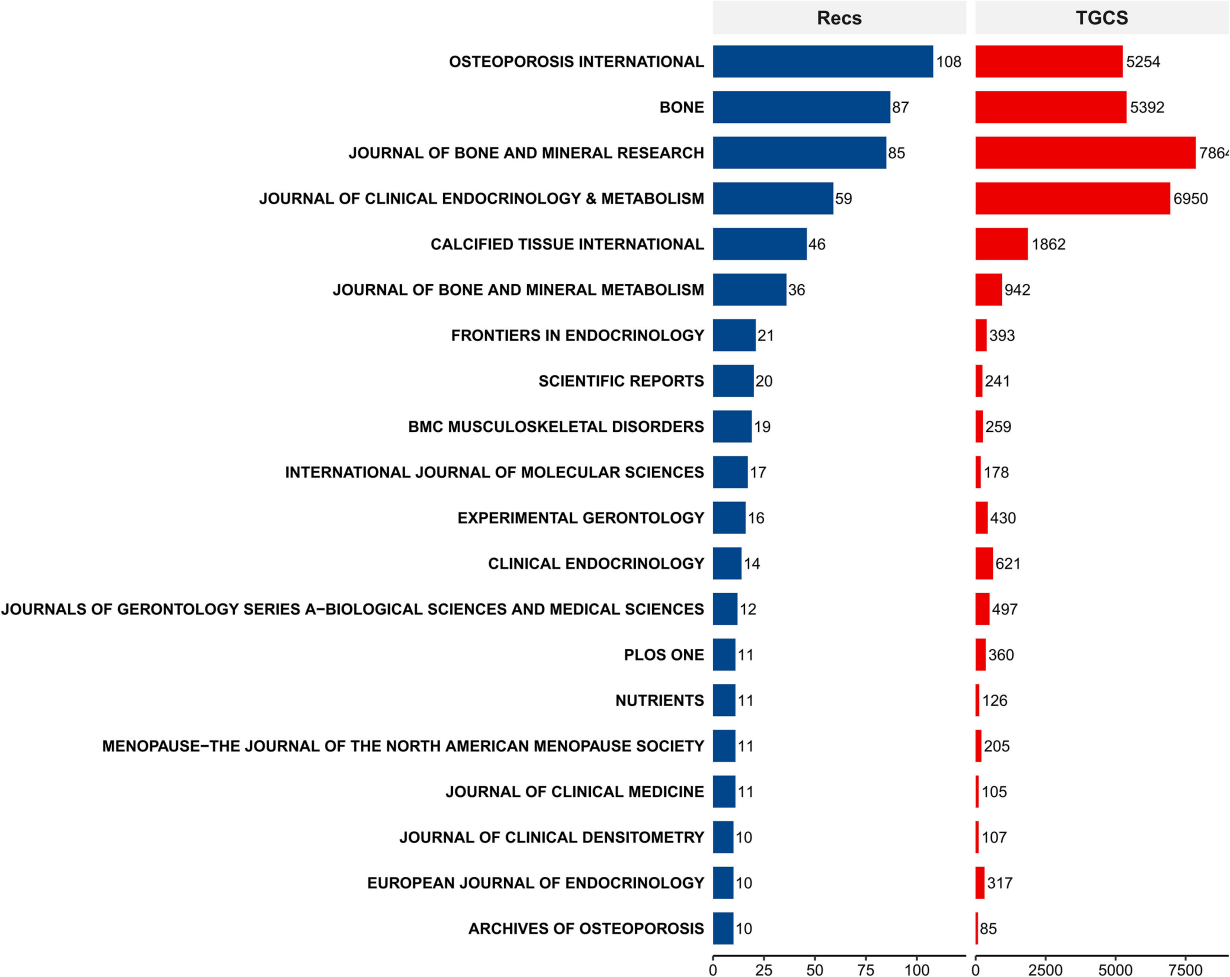
Bar chart of the top 20 core journals in OP biomarker research and their academic impact.

The retrieved literature was published across 601 academic journals. OP International (N = 108) and Bone (N = 87) emerged as the most prolific journals, followed closely by the Journal of Bone and Mineral Research (**Figure 3**). In terms of academic impact, the top 10 most cited articles were distributed across high-impact journals spanning multiple disciplines. These included Medicine & Science in Sports & Exercise, Progress in Biophysics & Molecular Biology, Journal of Clinical Endocrinology & Metabolism, and EBioMedicine, as well as authoritative orthopedic journals such as the Journal of Bone and Mineral Research and Bone.

### 3.3 Geographic distribution and collaboration networks

A total of 71 countries/regions participated in research within this domain, exhibiting a distinct regional concentration (**Table 1**). The United States secured the top position with 314 publications and a TGCS of 21,018, demonstrating a dual advantage in both quantity and quality. China (287 publications, TGCS: 5,050) and Japan (133 publications, TGCS: 3,883) ranked second and third, respectively. Notably, although Italy (129 publications) and the United Kingdom (119 publications) had slightly lower publication volumes, their TGCS reached 5,405 and 7,765, respectively. This observation suggests that research outputs from these nations possess exceptionally high average impact.

**Table 1:** Bibliometric indicators of the top 10 countries/regions in global OP biomarker research output.

Country	Recs	TGCS
United States	314	21018
China	287	5050
Japan	133	3883
Italy	125	5405
United Kingdom	119	7765
France	82	4817
Spain	76	2294
Germany	74	3829
Australia	68	3538
Denmark	53	2475

**Figure 4:**
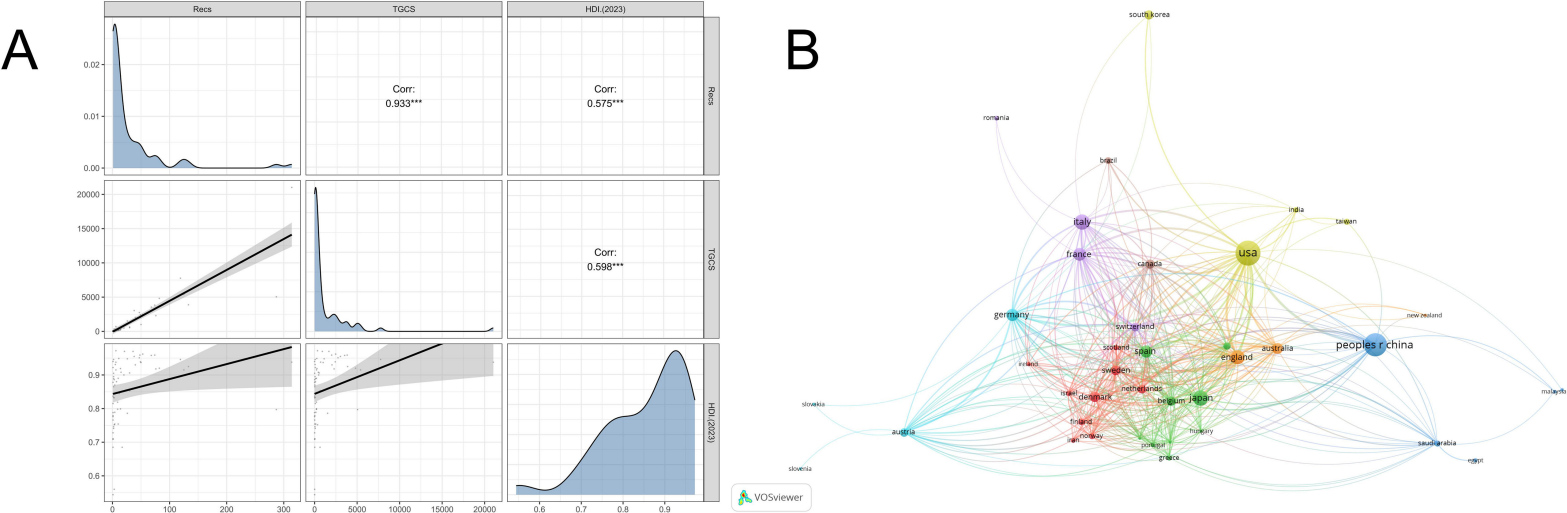
Geographic distribution and collaboration characteristics of global research output. **(A)** Spearman correlation analysis of publication counts, TGCS, and regional HDI. **(B)** Visualization of the international scientific collaboration network based on countries/regions.

Spearman correlation analysis revealed a significant positive correlation between publication volume/TGCS and the regional HDI. This indicates that a nation’s development level is closely linked to its research investment and impact in the field of OP biomarkers (**Figure 4A**). International collaboration network analysis (**Figure 4B**) further identified the United States, France, China, and Australia as core hub nodes within the global scientific collaboration network.

### 3.4 Keyword co-occurrence analysis

**Figure 5:**
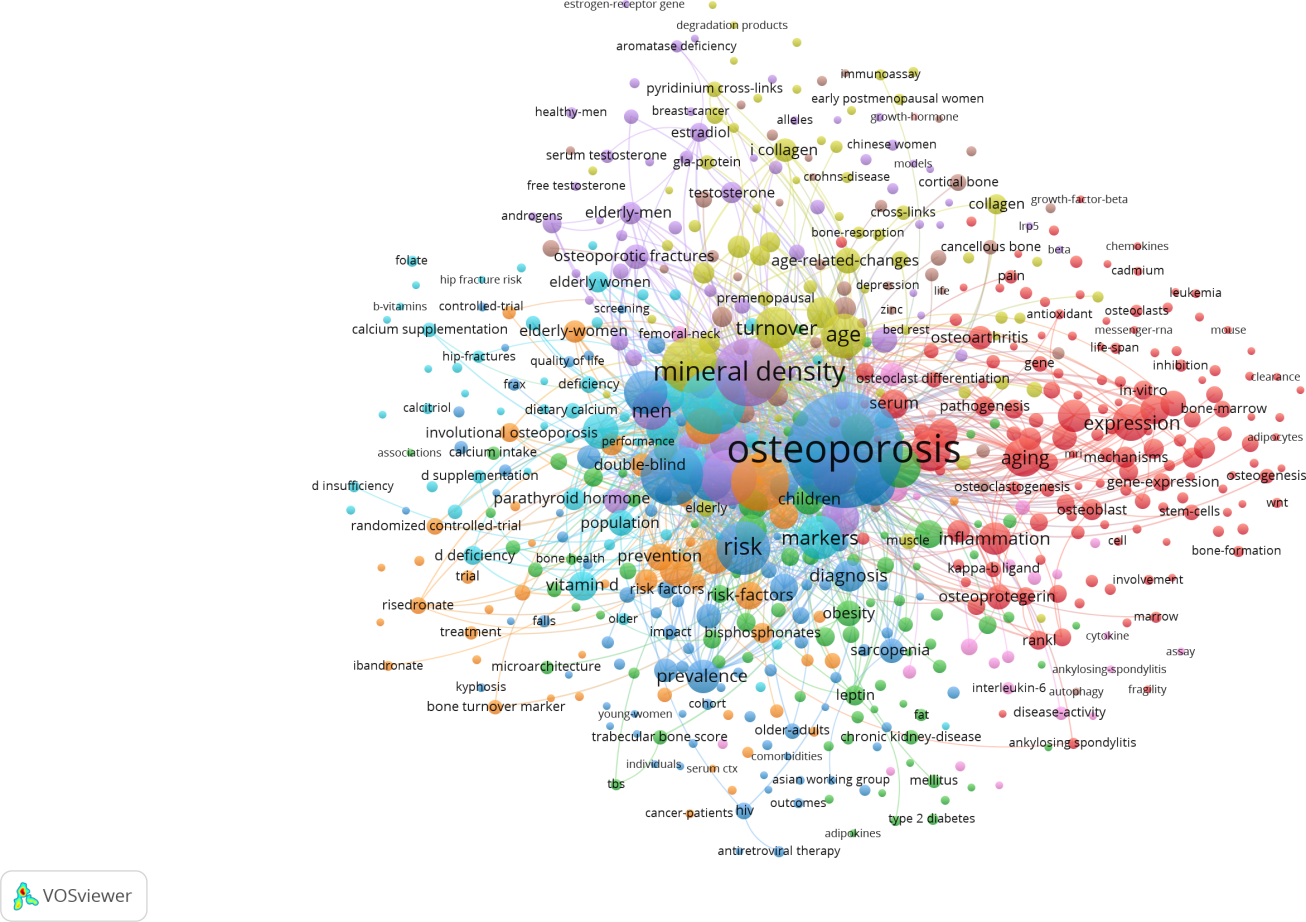
Keyword co-occurrence clustering network of OP biomarkers generated by VOSviewer.

Keyword clustering analysis was performed using VOSviewer, identifying nine core research theme clusters (**Figure 5**). These clusters are defined as follows: Cluster 1 (Red) represents bone metabolism, aging, and oxidative stress (keywords: Cellular senescence, Apoptosis, Aging); Cluster 2 (Green) involves the interaction between bone metabolism and chronic metabolic diseases (keywords: Obesity, Diabetes, CKD); Cluster 3 (Blue) pertains to fall risk and clinical prognosis (keywords: Mortality, Falls, Quality of life); Cluster 4 (Yellow) focuses on biochemical markers of bone turnover (keywords: Osteocalcin, CTX-I); Cluster 5 (Purple) relates to the sex hormone regulatory axis (keywords: Testosterone, Estradiol); Cluster 6 (Light Blue) concerns nutritional intervention and prevention (keywords: Vitamin D, Vitamin K); Cluster 7 (Orange) covers anti-osteoporotic pharmacotherapy (keywords: Denosumab, Bisphosphonates); Cluster 8 (Brown) addresses bone strength and imaging assessment (keywords: Finite element analysis, HR-pQCT); and Cluster 9 (Violet) signifies osteoimmunology (keywords: Osteoimmunology, Cytokines, Inflammation).

### 3.5 Identification of high-potential themes via strategic diagram

Quadrant analysis was conducted on embedded keyword clusters within the WoSCC and PubMed databases, utilizing the Walktrap algorithm and Callon’s strategic diagram. The study specifically focused on clusters situated in the fourth quadrant (Basic/Future Themes). Characterized by “high centrality and low density,” these themes represent potential directions that are closely associated with the core domain but have not yet fully matured.

**Figure 6:**
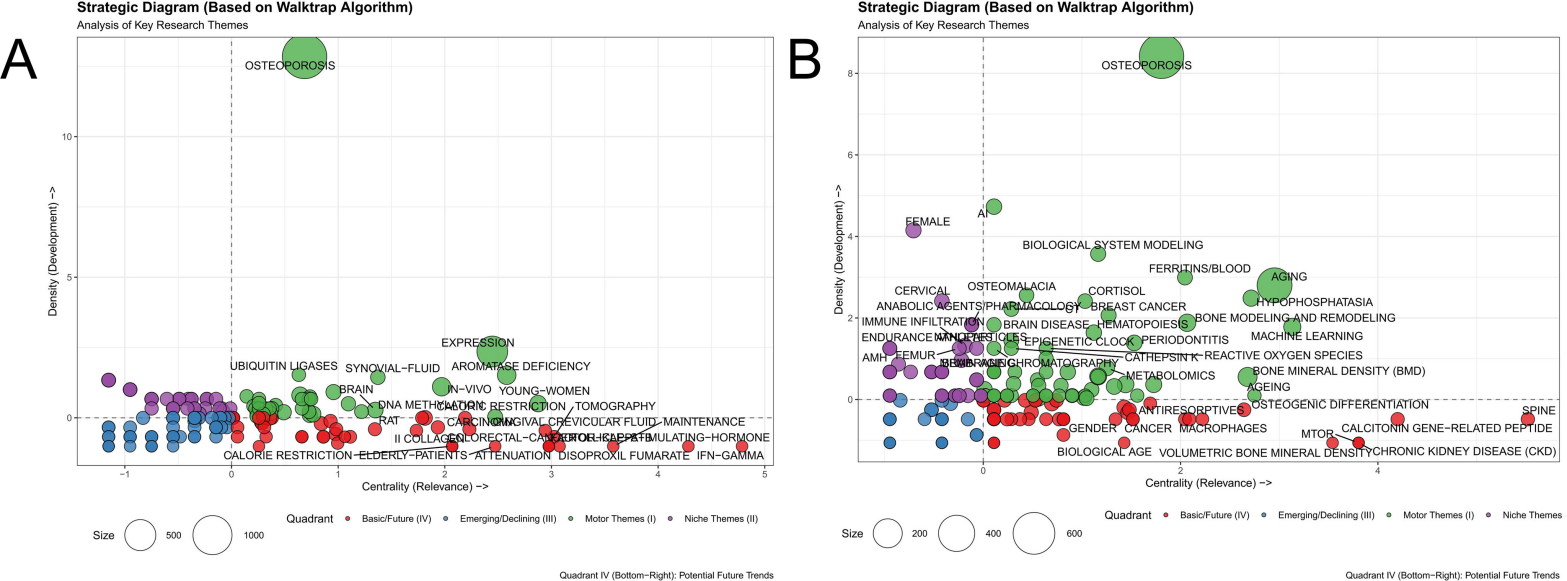
Identification of high-potential research themes based on the Walktrap algorithm and Callon’s strategic diagram. **(A)** Strategic coordinate analysis based on the WoSCC database. **(B)** Validation analysis based on the PubMed database.

In the WoSCC database, 27 clusters were identified in the fourth quadrant (**Figure 6A**). These primarily involved early diagnostic techniques (Early Diagnosis, Biosensing Techniques), immunoregulation (NLR Family, Regulatory T-Lymphocytes), and gut microecology (Gastrointestinal Microbiome, RNA 16S).

Similarly, validation analysis using the PubMed database identified 50 clusters located in the fourth quadrant (**Figure 6B**). These included the P38 MAPK pathway, Interleukin-1 metabolism, and plant extract therapy.

Through cross-validation and integration of results from both databases, three major high-potential fields were established: Inflammation and Immunoregulation: covering Cytokines, IL-1, P38 MAPK, NLRP3 Inflammasome, and Regulatory T cells (Tregs); Detection Techniques and Biosensing: covering Biosensing techniques, Electrochemical techniques, and Luminescent measurements; Gut Microecology and Metabolism: covering Gut microbiota, 16S rRNA, Bariatric surgery, and Short-chain fatty acids.

### 3.6 Construction of knowledge graph for high-potential fields

To ensure the clinical utility of the knowledge graph, a rigorous “Human-in-the-loop” validation protocol was implemented augmenting the LLM-based automatic extraction. While the LLM successfully extracted structured relationships, approximately 6.5% of the results were identified as “semantic noise”. This noise was manually filtered to guarantee high specificity in biomarker discovery.

**Figure 7:**
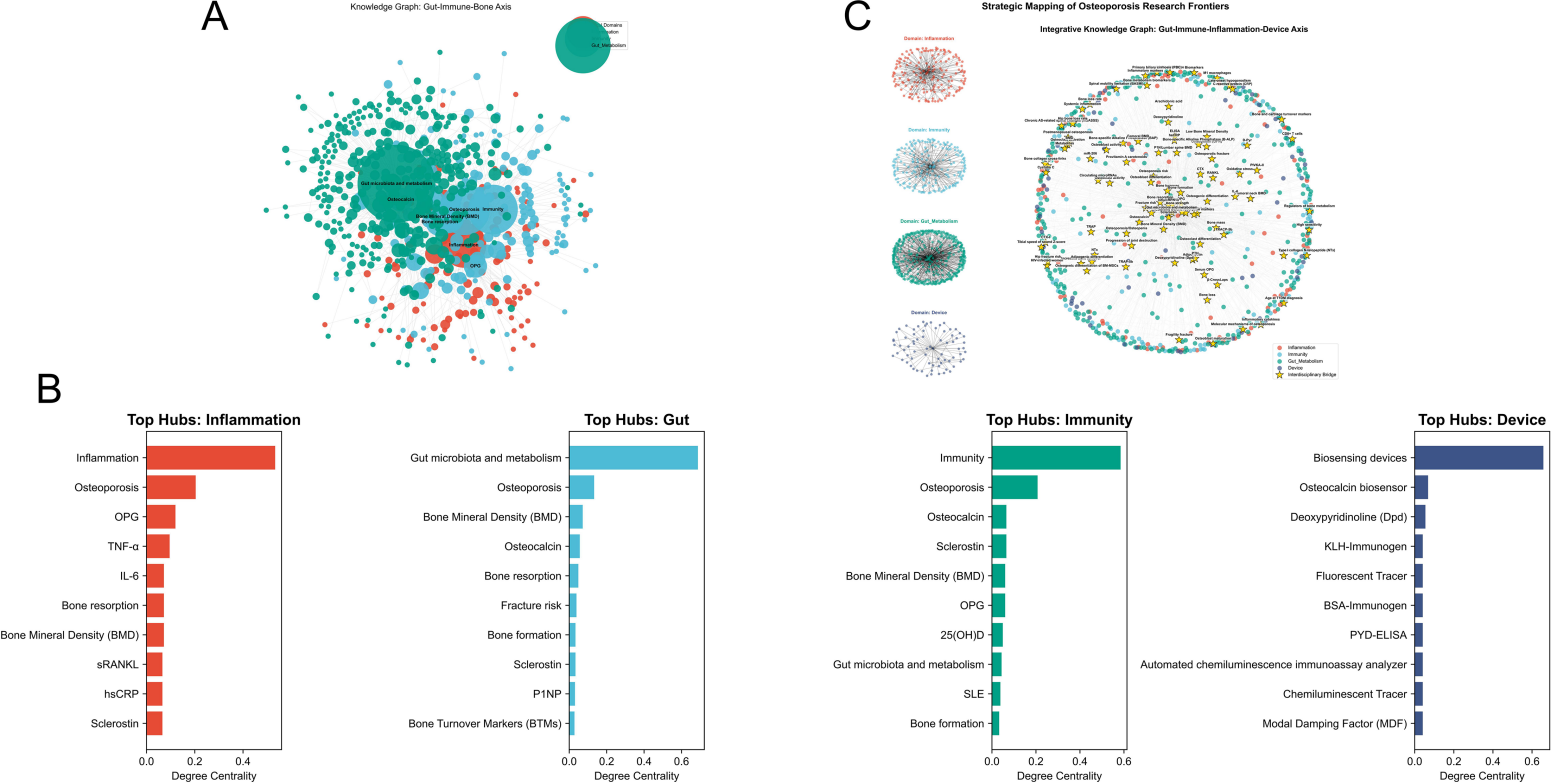
AI-driven construction and topological analysis of refined knowledge graphs in high-potential research fields. **(A)** Workflow diagram of knowledge graph construction using LLMs and the Human-in-the-loop mechanism. **(B)** Identification of key hub nodes in the four major domains: inflammation, immunity, gut microbiota and metabolism, and biosensing devices. **(C)** Cross-system bridging nodes and multidimensional interaction regulatory networks.

The data processing workflow proceeded as follows. First, duplicates within the bibliographic records from both databases were removed using NoteExpress. Subsequently, a full-text screening focused on the three high-potential fields was conducted, yielding 920 core articles selected from an initial pool of 1,577 relevant documents. Utilizing the Gemini model, 1,640 “entity-relationship” triplets were extracted. These comprised 361 triplets in the inflammation domain, 340 in immunity, 848 in gut microbiota and metabolism, and 91 in the field of biosensing devices (**Figure 7A**).

Based on centrality analysis, key nodes were identified within each domain. In the inflammation domain, key nodes included Inflammation, OP, OPG, TNF-α, IL-6, Bone resorption, Bone Mineral Density (BMD), sRANKL, hsCRP, and Sclerostin. In the immunity domain, identified nodes comprised Immunity, OP, Osteocalcin, Sclerostin, BMD, OPG, 25(OH)D, Gut microbiota and metabolism, SLE, and Bone formation. Within the gut microbiota and metabolism domain, key nodes included Gut microbiota and metabolism, OP, BMD, Osteocalcin, Bone resorption, Fracture risk, Bone formation, Sclerostin, P1NP, and Bone Turnover Markers. Finally, in the biosensing devices domain, key nodes included Biosensing devices, Osteocalcin biosensor, Deoxypyridinoline, KLH-Immunogen, and Fluorescent Tracer (**Figure 7B**).

The construction of the knowledge graph revealed common bridge nodes linking different domains. Oxidative stress (centrality: 0.215) bridged immunity, inflammation, and gut microbiota and metabolism. RPN1 (centrality: 0.128) connected immunity with gut microbiota. PIVKA-II (centrality: 0.176) linked gut microbiota with biosensors. Circulating microRNAs (centrality: 0.035) and miR-206 (centrality: 0.054) connected inflammation and immunity. Furthermore, Arachidonic acid (centrality: 0.051), Cystatin C (centrality: 0.073), Leptin (centrality: 0.059), Provitamin A carotenoids (centrality: 0.059), and Adiponectin (centrality: 0.058) served as bridges between inflammation and gut microbiota (**Figure 7C**).

Subsequently, nodes exhibiting a Degree below the median (indicating they are not current research hotspots) but ranking high in Betweenness centrality were designated as high-potential nodes. In the inflammation domain, 41 emerging potential nodes were identified, including lncRNA HOTAIR, lncRNA MEG3, circ-Trn4, miR-21-5p, Serum Chemerin, Vaspin, Visfatin, HMGB1, IL-33, and PTX3. In the immunity domain, 38 emerging potential nodes were identified, such as Treg-Exo, Macrophage polarization, and Th17/Treg balance. In the gut microbiota and metabolism domain, 36 potential nodes were identified, including Vertebral bone marrow PDFF, Glutamine, and S-equol. Finally, in the biosensing devices domain, 27 potential nodes were identified, including Quantitative Susceptibility Mapping, Opportunistic CT, Deep learning automated CT segmentation, 41Ca and Accelerator Mass Spectrometry, Raman spectroscopy, Chip-based microfluidic capillary electrophoresis system, Label-free immunosensor for CTX, and Bone-targeted engineered EVs.

## 4 Discussion

This study innovatively employed the AI embedding model (text-embedding-3-large) combined with complex network algorithms to systematically reconstruct the knowledge graph of global OP biomarker research. Distinct from traditional bibliometrics that focus solely on explicit hotspots, this research successfully identified potential frontier themes located in the “fourth quadrant” (high centrality, low density) through deep semantic clustering in a high-dimensional vector space. These findings reveal that bone metabolism research is undergoing a paradigm shift from “single hormone regulation” to a “multi-system interaction network” (involving inflammation, immunity, gut microecology metabolism, and imaging). This provides solid evidence-based grounds for the development of next-generation precision diagnosis and treatment strategies.

The emerging nodes identified in this study, such as lncRNA HOTAIR, MEG3, and miR-21-5p, highlight the core regulatory role of non-coding RNAs within the bone remodeling microenvironment. lncRNA MEG3 was identified as a key negative regulator of osteogenic differentiation in bone marrow mesenchymal stem cells (BMSCs). In patients with postmenopausal OP and model mice, MEG3 expression is significantly upregulated, inhibiting the osteogenic process by targeting the miR-133a-3p/SLC39A1 axis. Based on this mechanism, recent nanomedicine research has developed engineered exosomes loaded with magnetic nanoparticles. By targeted silencing of MEG3 or its downstream molecules, these interventions successfully achieved significant improvements in bone microstructure (BV/TV, Tb.N) in diabetic OP models (16–18).

As highly enriched cargo within exosomes, miR-21-5p demonstrates potential for cross-cellular regulation. On one hand, miR-21-5p secreted by BMSCs can target and inhibit the negative regulator KLF3, thereby enhancing osteogenic activity. On the other hand, miR-21-5p derived from adipose-derived stem cells promotes angiogenesis and accelerates bone defect repair via the NOTCH1/DLL4/VEGFA axis (19–21). This indicates that miR-21-5p holds promise as a dual-effect nucleic acid drug target capable of promoting both osteogenesis and vascularization.

Functioning as a bridge between nuclear epigenetic silencing and cytoplasmic post-transcriptional regulation, the function of HOTAIR is highly dependent on its subcellular localization. Within the nucleus of BMSCs, HOTAIR acts as a molecular scaffold to recruit the PRC2 complex (e.g., Ezh2). This increases H3K27me3 levels in the promoter regions of key osteogenic genes (Runx2, Sp7) and suppresses the Wnt/β-catenin pathway, thereby hindering osteogenic differentiation (22, 23). Interestingly, upon translocation to the cytoplasm, it transforms into a competitive endogenous RNA (ceRNA). Through “sponging” miR-214, it relieves the inhibition of Atf4, thus promoting bone formation (23, 24). This nucleocytoplasmic shuttling mechanism suggests that the expression level and subcellular distribution pattern of HOTAIR may serve as sensitive indicators reflecting the dynamics of bone cell injury and repair.

In the field of osteoimmunology, regulatory T cell-derived exosomes (Treg-Exo) were identified as a critical “bridge node.” The surface of Treg-Exo is rich in CD39 and CD73 nucleotidases, which convert ATP in the inflammatory environment into adenosine, an immunosuppressive molecule. This process induces macrophage polarization toward the anti-inflammatory phenotype (M2) and promotes the recruitment and proliferation of MSCs. Compared to traditional stem cell transplantation, Treg-Exo offers advantages such as low immunogenicity, absence of tumorigenic risk, and ease of engineering modification. Preclinical studies have demonstrated that hydrogel systems loaded with Treg-Exo significantly accelerate the healing process of complex fractures (25–28). This discovery marks a shift in OP treatment strategies from “cell replacement” to safer “cell-free regenerative therapy”.

Topological analysis revealed that Serum Spermidine possesses extremely high betweenness centrality, suggesting that the polyamine metabolic network plays a key intermediary role in skeletal health. Evidence from high-resolution metabolomics indicates that spermidine not only prevents bone loss via the autophagy pathway but that elevated levels of its metabolite, N-acetylputrescine, are also significantly negatively correlated with spinal BMD (29, 30).

Furthermore, the identification of Vertebral bone marrow PDFF (Proton Density Fat Fraction) underscores the pathological significance of the transformation of the bone marrow microenvironment from “hematopoietic/osteogenic tissue” to “adipose tissue.” Bone marrow adipose tissue acts not merely as a filler but as an active endocrine organ. PDFF accurately quantifies the degree of bone marrow fat infiltration. Its AUC for diagnosing OP reaches 0.925, superior to bone mass measurement alone, making it an early sensitive indicator reflecting the deterioration of the bone marrow microenvironment (31–33).

Addressing the issue of low screening rates with traditional DXA, the “Opportunistic CT” combined with deep learning technology mined in this study represents a future direction for screening. Algorithms such as VB-Net or CNNs can automatically segment bones and extract radiomics features from routine abdominal or chest CT scans. This achieves high-precision (AUC > 0.960) opportunistic screening for OP without additional radiation exposure or cost (34, 35). Simultaneously, the emergence of Quantitative Susceptibility Mapping technology resolves the difficulty of quantifying bone minerals with MRI, opening a new path for “radiation-free, high-precision” bone quality assessment.

In our integrative knowledge graph, several key nodes such as lncRNA HOTAIR, miR−21−5p, and OPG have been implicated in bone remodeling, osteoclast/osteoblast balance, and OP pathogenesis. Long noncoding RNA HOTAIR has been shown to regulate osteogenic differentiation of bone marrow mesenchymal stem cells and modulate osteoblast activity, suggesting its potential as both a biomarker and therapeutic target in OP and impaired bone formation (24, 36, 37). MicroRNA miR−21−5p has been experimentally demonstrated to inhibit osteoclastogenesis and attenuate bone resorption in ovariectomized mouse models, indicating its potential utility for mitigating bone loss and serving as a therapeutic (38) target. OPG, a decoy receptor for receptor activator of nuclear factor−κB ligand (RANKL), is a key regulator of osteoclast differentiation, and alterations in the OPG/RANKL axis are associated with clinical measures of bone turnover and OP progression (39). Together, these bridge and hub nodes not only occupy central computational roles in our network but also show translational relevance as candidate biomarkers or therapeutic targets, providing mechanistic insight and avenues for further clinical investigation.

Synthesizing the above findings, the potential nodes proposed in this study constitute the three pillars of future precision medicine for OP: (1) Early warning: Liquid biopsy markers represented by circRNA, miR-21-5p, and spermidine, used to capture early molecular changes in the bone microenvironment; (2) Precision phenotyping: Utilizing PDFF and Quantitative Susceptibility Mapping to achieve multi-dimensional quality assessment of “bone density-bone marrow fat-bone microstructure”; (3) Targeted therapy: Using Bone-targeted engineered EVs as carriers to achieve precise delivery of nucleic acid drugs to lesions. Notably, the discovery of nodes such as S-equol further corroborates the importance of the “gut-bone axis,” suggesting that regulating the metabolic capacity of the gut microbiota may be a new strategy for intervening in postmenopausal OP.

Despite the employment of advanced AI embedding models, this study retains certain limitations. First, bibliometrics possesses an inherent lag; thus, some recent breakthrough studies may not have been fully recognized due to insufficient citation accumulation. Second, although LLMs were introduced for semantic correction, the standardization of interdisciplinary terminology still requires broader expert consensus. Future research should aim to integrate multi-omics raw data (e.g., GEO/TCGA) and conduct wet-lab validation of the emerging nodes identified in this study to accelerate their clinical translation process.

Our integrative knowledge graph highlights candidate biomarkers and regulatory molecules that may guide OP diagnosis and therapy. For instance, bridge nodes with high degree centrality such as lncRNA HOTAIR and miR−21−5p can be prioritized for experimental validation in preclinical models. OPG and other hub proteins identified may serve as indicators for fracture risk or treatment response. Future studies should aim to correlate these network-derived candidates with patient cohorts, assess predictive value for bone density and fracture risk, and evaluate intervention strategies in clinical trials. This translational approach will help bridge computational insights with practical clinical applications.

## Data Availability

The original contributions presented in the study are included in the article/**Supplementary Material**. Further inquiries can be directed to the corresponding author.
